# Reviewed article: Proença R, Nunes CCMN, Romano APM, Silva CP, Frank CHM, Moraes FS, Gonçalves FSS, Bandeira GS, Rodrigues IA, Cavalcante JR, Fonseca JLP, Silva LC, Duarte MMS, Maia MA, Mello MS, Nascimento MP, Nienov OH, Costa POM, Martins RCC, Melo SA, Ferreira VP, Casaroto VS, Freitas DRC, Almeida TVR, Garcia MHO, Barreira Filho EB. National Guidelines for Public Health Emergency Surveillance within the Brazilian Unified Health System. Epidemiol Serv Saude. 2026:35;e20260071

**DOI:** 10.1590/S2237-96222026v35e20260071.b

**Published:** 2026-10-12

**Authors:** Cristine Bonfim

**Affiliations:** 1 Fundação Joaquim Nabuco, Recife, PE, Brazil

## Peer review B

## Questionnaire

Does the manuscript contain new and significant information to justify publication? Yes

Does the Abstract (Summary) clearly and accurately describe the content of the article? No

Is the problem significant and concisely stated? No

Are the methods described comprehensively? Not applicable

Are the interpretations and conclusions justified by the results? Not applicable

Is adequate reference made to other work in the field? No

## Manuscript Structure

Length of article is: Adequate

Number of tables is: Too few

Number of figures is: Too few

Please state any conflict(s) of interest that you have in relation to the review of this paper. None

Interest: Good

Quality: Good

Originality: Good

Overall: Good

Recommendation: Major Revision

## Comments

Comentários maiores

Entende-se que se trata de um artigo especial, por esse motivo a estrutura do objetivo não é a convencional da RESS. No entanto, o conteúdo dos itens citados não condiz com os títulos das seções do resumo. Talvez se o resumo tivesse um caráter de apresentação, ficasse mais adequado.

Embora, se trate de um artigo especial na modalidade “Guidelines”, ainda é uma publicação científica e será disponibilizado na RESS. Importante que o manuscrito busque um diálogo com a literatura científica, em várias passagens as afirmativas podem e devem ser subsidiadas por essa literatura. Os documentos institucionais são necessários para o guia, mas em várias passagens podem ser substituídos pela literatura científica.

Seria interessante e oportuno que o guia também incluísse exemplos, sobre a atuação das vigilâncias citadas. Além disso, uma tabela com os tipos de vigilância e as portarias que regem cada uma delas, traria uma ótima sistematização para os(as) leitores(as).

Necessidade da utilização de quadros para uma sistematização e consulta rápida das informações.

O último parágrafo da seção de aplicabilidade ao SUS, de fato não é uma aplicação e sim um desafio. Seria interessante acrescentar nessa seção os desafios ou os limites das vigilâncias.

A conclusão precisa responder ao objetivo do guia.

Observa-se um excesso de referências institucionais, mesmo para o tipo de artigo. Além do pouco diálogo com a literatura científica.

Comentários menores

O título

Seria interessante incluir que são diretrizes para o Brasil, no âmbito do SUS e o ano ou período. Para que ocorra uma perfeita harmonia com o objetivo proposto.

Objetivo

Sugere-se substituir apresentar por descrever.

Descrever as diretrizes para a vigilância das emergências em saúde pública no âmbito do Sistema Único de Saúde (SUS) no Brasil, período.

Diretrizes

A primeira frase pode ser removida, pois é uma explicação e não uma diretriz.

As frases podem ser mais curtas e diretas, além de evitar a repetição do termo vigilância.

O item discussão de fato não traz uma discussão. Consiste muito mais em uma operacionalização da vigilância.

Página 2, linha 8 - incluir as referências que fundamentam tal afirmativa.

Página 2, segundo parágrafo - a referência 3, pode ser substituída por artigos científicos.

Página 2, terceiro parágrafo - podem ser incluídas referências de artigos científicos que abordem a vigilância.

Página 2, quarto parágrafo - incluir referências de artigos científicos internacionais e nacionais que tratem sobre a vigilância das emergências em saúde pública.

Página 2, último parágrafo - Incluir a referência que fundamenta a afirmativa.

Página 3, primeiro parágrafo da seção de fundamentação conceitual - necessita de referências científicas que fundamentem as afirmativas.

A última frase dessa seção está solta e pouco compreensível. Qual temática?

Tabelas

A tabela 1 - traz conceitos não expostos no texto, além disso seria importante incluir alguma forma de sistematização das vigilâncias relatadas.

